# Reduction of Ochratoxin A during the Preparation of Porridge with Sodium Bicarbonate and Fructose

**DOI:** 10.3390/toxins13030224

**Published:** 2021-03-19

**Authors:** Hyun Jung Lee, Shufang Li, Kejia Gu, Dojin Ryu

**Affiliations:** 1 Department of Animal, Veterinary, and Food Sciences, University of Idaho, 875 Perimeter Drive MS 2330, Moscow, ID 83844-2330, USA; 2 Institute of Quality Standards and Testing Technology for Agro-Products, Henan Academy of Agricultural Science, Zhengzhou 450002, China; lishufang@hnagri.org.cn; 3 School of Food Science, Washington State University, P.O. Box 646376, Pullman, WA 99164-6376, USA; Kejia.gu@wsu.edu

**Keywords:** Ochratoxin A (OTA), indirect steaming, sodium bicarbonate, fructose

## Abstract

Ochratoxin A (OTA) is a potential human carcinogen that poses a significant concern in food safety and public health. OTA has been found in a wide variety of agricultural commodities, including cereal grains. This study investigated the reduction of OTA during the preparation of rice- and oat-based porridge by a simulated indirect steam process. The effects of sodium bicarbonate (NaHCO_3_) and fructose on the reduction of OTA were also investigated. During the processing, OTA in rice- and oat-porridge was decreased by 59% and 14%, respectively, from initial OTA artificially added at 20 μg/kg (dry weight basis). When 0.5% and 1% of sodium bicarbonate were added to rice porridge, increased reduction of OTA was observed as 78% and 68%, respectively. The same amounts of added sodium bicarbonate also further reduced OTA in oat porridge to 58% and 72%, respectively. In addition, increased reduction of OTA in the presence of fructose was observed. A combination of the two, i.e., 0.5% sodium bicarbonate and 0.5% fructose, resulted in a 79% and 67% reduction in rice porridge and oat porridge, respectively. These results indicate that indirect steaming may effectively reduce OTA in preparation of porridge-type products, particularly when sodium bicarbonate and/or fructose are added.

## 1. Introduction

Oats (*Avena sativa*) have been cultivated mainly in temperate regions worldwide as an important part of the human diet for millennia and are used in the production of various foods, including breakfast cereals, infant cereals, and snacks. Oats have been gaining popularity in recent decades due to their health benefits, such as reducing cardiovascular diseases and promoting immune functions driven by soluble fibers, including β-glucans [1,2,3]. Meanwhile, higher incidences and levels of ochratoxin A (OTA) have been reported from oats and oat-based products compared to other cereal grains such as wheat and corn and their products [4,5,6,7,8].

OTA is one of the major naturally occurring foodborne mycotoxins and is produced by a number of *Aspergillus* and *Penicillium* species. OTA is a possible human carcinogen in Group 2B with sufficient evidence of carcinogenicity in experimental animal studies [9,10]. In addition to its prominent nephrotoxicity, OTA is known to be hepatotoxic, teratogenic, mutagenic, and immunosuppressive [10,11,12]. Moreover, public health concerns are increasing worldwide as OTA frequently contaminates many commodities, including nuts, spices, dried fruits, and most cereal grains around the globe [13,14,15,16].

The majority of previous reports on the heat stability of OTA are in agreement that the complete reduction of OTA may not be achieved by conventional methods of food processing [17,18,19,20,21,22,23,24]. Steaming is a major food process that uses water vapor or heat, accompanied by the correct degree of temperature, and pressure as well sometimes. There are two kinds of steaming methods—“low-pressure steaming,” also called indirect steaming in this manuscript, and “high-pressure steaming,” such as direct steam injection (DSI) or retorting. While low-pressure steaming is a process where food is steamed through neither indirect nor direct contact with the steam, high-pressure steaming requires some equipment or containers that will not enable steam and/or heat to escape from food. Due to the major benefit of steaming that nutrients such as Vitamin C and B will not be lost from applying this method, steaming is one of the most popular food processes to make grain porridge products for the old and the infirm, who are more vulnerable to the toxic effects of OTA than healthy people. Since each liter of water at 100 °C requires approximately 2250 KJ or 5.35 times more energy to make dry steam than it does to raise the same amount of water from 0 °C to 100 °C [25], high-pressure steaming provides very quick heat transfer and could be effective in reducing OTA in foods despite the short processing time [19,24]. Unlike high-pressure steaming, such as DSI and retorting, [19,24], the effects of low-pressure steaming, such as indirect steaming, on the reduction of OTA have not been studied so far. In this study, two distinctively different matrices, i.e., rice and oats with different compositions including fiber content, were employed to study the heat stability of OTA during a simple thermal process of indirect steaming. As a practical measure to enhance the reduction of OTA, the effects of adding sodium bicarbonate and fructose were also investigated.

## 2. Results

The color of food products is an important yet simple and fast quality indicator, as it can serve as an indicator of physicochemical properties, including the amount of pigments [26]. As shown in Table 1, the addition of sodium bicarbonate (NaHCO_3_) affected the color of rice- and oat-based porridges significantly. Decreased lightness (*L*) with added sodium bicarbonate was observed in both rice and oat porridges after the cooking process (*p* < 0.05), while the redness (*a*) of the porridges increased significantly (*p* < 0.05). When sodium bicarbonate was added, increased yellowness (*b*) was observed in the cooked rice porridge but no significant changes in oat porridge. With added fructose, only rice porridge showed an increasing trend in yellowness (*b*).

The analytical method for OTA employed in this study was verified with the recovery ranged 79–92% for rice- and 104–111% for oat-based samples. In addition, the limit of detection (LOD) and limit of quantification (LOQ) for rice- and oat-based samples were 0.032 μg/kg and 0.10 μg/kg, 0.038 μg/kg and 0.12 μg/kg, respectively. The values of LOD and LOQ were determined with their respective signal-to-noise ratio, i.e., 3 and 10.

Rice and oats were hired for the current study in an attempt to explain greater frequency and levels of OTA in oat-based products in comparison with other cereal-based processed foods [4,5,6,7,27]. During the cooking process at 80–85 °C of center temperature, 59.4% and 13.6% reduction of OTA concentration was observed in rice- and oat- porridge, respectively. Moreover, the effects of sodium bicarbonate and fructose on OTA during thermal processing in a simulated indirect steaming process were demonstrated with the two different matrices of rice and oats (Table 2).

In the two food matrices, the amount of added sodium bicarbonate showed varying effects on the reduction of OTA observed (Table 2). When compared with the reduction of the toxin in rice porridge cooked with no additive (59.4%), the addition of 0.5% and 1.0% sodium bicarbonate resulted in the reduction of 78.1% and 68.7%, respectively. On the other hand, added sodium bicarbonate made a greater impact in oat porridge by increasing the reduction of OTA from 13.6% (no additive control) to 57.7% and 72.6% with 0.5% and 1.0% sodium bicarbonate, respectively.

The addition of fructose also resulted in a significant reduction of OTA in both matrices depending on the amount (Table 2). When fructose was added at 0%, 0.5%, 1%, 5%, and 10% (*w/w*) in this study, observed OTA reductions in rice porridge were 59.4%, 62.5%, 80.7%, 66.1%, and 60.0%, respectively, while the reductions in oat porridge were more evident with 13.6%, 47.3%, 69.3%, 47.5%, and 40.7%, respectively. It may be noted that adding fructose at 1% resulted in the highest reduction of OTA in both food matrices then gradually decreased with the increasing amount of added fructose. Although observed reduction was greater in rice porridge, i.e., 80.7% reduction at the peak, actual impact or benefit of adding fructose seemed greater in oat porridge as 55.7% more OTA was reduced with 1% fructose in comparison with the no sugar added control, i.e., from 13.6% to 69.3%.

## 3. Discussion

It is well known that OTA is very heat stable compared with other mycotoxins, while significant reduction of OTA may be observed under alkaline conditions or high temperature with high pressure, such as extrusion [17,19,21,28,29]. In order to increase pH or achieve alkaline conditions in food, using sodium bicarbonate or baking soda would be the only feasible option as it is generally recognized as safe or GRAS [30]. In the case of sugar as an additive, its effect on the reduction of toxicity of OTA during food processing has not been studied, while adding sugars reduced the amount and toxicity of other mycotoxins, such as fumonisin B_1_ during extrusion [31,32]. Even though the chemical structures of fumonisin B_1_ and OTA are different, we investigated the possibility using three different sugars of glucose, fructose, and sucrose and observed increased reduction of OTA during thermal processing by adding fructose [33]. According to the study [33], adding fructose resulted in a significantly lower OTA level than glucose, sucrose, or no-sugar-added samples. At the side of toxicity, moreover, the addition of fructose resulted in an increased OTA degradation products profile to less toxic OTα-amide, not only significantly reduced the OTA level [33]. Contrastively, an increased OTA isomer level, which has similar toxicity to OTA, was produced by adding glucose, sucrose, or no sugar [33]. Previously, Lee Gu, Ganjyal, and Ryu [19] reported a 19.8% and 27.9% reduction of OTA in oat slurry during a DSI process operated at 85 °C and 121 °C, respectively. Other studies also suggested the role of matrices on the reduction of OTA at varying extents. Ryu, Kowalski, Ganjyal, and Lee [21] demonstrated that OTA reduction in rice flour ranged 77.9–82.2% while the toxin in oat flake was reduced by 39.5–42.7% during the same processing conditions of a laboratory-scale twin-screw extruder. During autoclaving of oatmeal and rice cereal for up to 3 h, the reduction of OTA ranged from 86.0% to 87.5% while the extent of reduction was decreased to 74.0% and 68.5% when the samples were autoclaved with 50% (*v*/*w*) water [34]. Another simple food processing method of explosive puffing under varying pressures ranging from 0.5 to 0.9 MPa resulted in greater reduction of OTA in oats (37.7–52.2%) in comparison with the reduction in rice (15.3–28.4%) [20].

Meanwhile, specific heat may help understanding the matrix effect as it equals to the sum of pondered contribution of each component including carbohydrates, protein, lipids, salts, and water. While the specific heat of water is 4.18 kJ/kg or 1 cal/g, the specific heat of the major components is taken as: salts = 0.2; carbohydrate = 0.34; protein = 0.37; and lipids= 0.4 relative to water [35]. According to USDA FoodData Central [36], oat flour (100 g) contains 13.33 g of protein; 6.67 g of lipids; 73.33 g of carbohydrate, while rice flour (100 g) contains 3.57 g of protein; 0.89 g of lipids; 80.36 g of carbohydrate. Since specific heat is different by component of food matrix, its heat transfer or heat energy can affect OTA differently in different matrices.

The amount of added sodium bicarbonate showed varying effects on the reduction of OTA in the two food matrices (rice and oat) observed (Table 2). It may be noted that the increased amounts of added sodium bicarbonate did not always correspond to a greater reduction of OTA as similar trends were observed during the extrusion of rice and oats [21]. During the extrusion of oat flakes, the addition of sodium bicarbonate at 0.5% and 1.0% resulted in 57% and 65% reduction, respectively, compared to 41% reduction with no added sodium bicarbonate. In contrast, during the extrusion of rice flour, a lesser reduction of OTA was observed with higher amounts of added sodium bicarbonate, i.e., 75–80% with 0.5% and 72–77% reduction with 1.0% sodium bicarbonate, in comparison with 78–82% reduction of OTA without sodium bicarbonate [21]. Meanwhile, a previous study by Lee, Gu, Ganjyal, and Ryu [19] showed a dose-dependent reduction of OTA during DSI processing of oat-based infant cereals. At 121 °C of DSI, the reduction of OTA was increased from 27.9% (no additive control) to 44.3% and 51.4% with 0.5% and 1.0% sodium bicarbonate, respectively, while the same processing at lower temperature of 85 °C also resulted in similar reductions, i.e., from 19.8% (no additive control) to 36.1% and 43.4% with 0.5% and 1.0% sodium bicarbonate, respectively [19].

Components or ingredients in the food matrix including sugars may undergo various reactions during processing to result in color changes and production of volatile compounds. Thermal processing of sugars or carbohydrates may also cause alterations in pH to yield organic acids, such as lactic acid and formic acid [37,38,39,40]. With its higher carbohydrate content, rice can produce more organic acids than oats that may positively contribute to the stability of OTA, i.e., acidic conditions. Dahal, Lee, Gu, and Ryu [17] demonstrated previously that OTA is more stable under acidic conditions during thermal processing. Trenk, Butz and Chu [34] also showed lesser OTA reduction in the presence of acetic acid during autoclaving of rice and oatmeal. When high-pressure steaming, such as retorting, was applied to reduce OTA levels with fructose alone or a combination of fructose and sodium bicarbonate [24], the reduction trends by adding additives were different trends with low-pressure steaming, such as indirect steaming used in this study. According to [24], the greater OTA reduction in retorted oat-based porridges was observed by adding fructose (no additives 17.2%, 0.5% fructose 40.8%, 5% fructose 35.5%, respectively), while the decreased OTA reduction in retorted rice-based porridges was observed by increasing amount of fructose (no additives 53.8%, 0.5% fructose 38.7%, 5% fructose 18.2%, respectively). Such observation may be corroborated with a similar extent of OTA reduction between the two groups of fructose alone (0.5%) vs. the combination of fructose and sodium bicarbonate (0.5% + 0.5%) suggesting that the mechanisms of the two additives are not in common. Lee, Lee, and Ryu [24] also reported that the reduction of OTA in retorted oat- and rice-based porridges was similar values with 0.5% fructose treatment when combined with 0.5% sodium bicarbonate and 0.5% fructose was added during the retorting process. This is the first study reporting OTA reduction during the manufacturing of common porridge products with added fructose and sodium bicarbonate. These data confirmed that the food matrix can affect the OTA reduction while added fructose or sodium bicarbonate can facilitate the reduction of OTA possibly by changing its pH conditions. Nonetheless, the decreased OTA concentration determined by HPLC in this study may not correspond to reduced toxicity as varying or unknown degradation products can be formed during the process. Therefore, further research is required to prove the loss of toxicity and to elucidate the mechanism of reaction between OTA and the additives during thermal processes including indirect steaming.

## 4. Conclusions

Simulated indirect steam processing of porridges resulted in significant reductions of OTA, i.e., 59.4% in rice porridge and 13.6% in oat porridge. The addition of sodium bicarbonate significantly influenced the reduction of OTA with up to 78.1% reduction in rice porridge with 0.5% sodium bicarbonate and 72.6% reduction in oat porridge with 1% sodium bicarbonate. Among all levels of added fructose, the highest reduction of OTA resulted from 1% fructose (*w/w*) with up to 80.7% in rice porridge and 69.3% in oat porridge. Similar to the samples treated with 1% fructose, adding a combination of 0.5% fructose and 0.5% sodium bicarbonate increased the reduction of OTA in rice porridge and oat porridge up to 78.6% and 67.2%, respectively, in comparison with the porridges without additives. These results indicate that OTA indirect steaming of cereal-based porridge may reduce OTA significantly particularly when sodium bicarbonate and/or fructose are added. For commercial application, additional studies on the residual toxicity need to be performed as the reaction or degradation mechanism of OTA during the indirect steaming process is largely unknown including its degradation products and their toxicity.

## 5. Materials and Methods

### 5.1. Chemicals and Materials

Food-grade oat flour was obtained from the Grain Millers, Inc. (Eugene, OR, USA), and food-grade rice flour was obtained from Bob’s Red Mill Natural Foods (Milwaukee, OR, USA). Food-grade fructose and sodium bicarbonate were purchased from Now Foods (Bloomingdale, IL, USA), Church & Dwight Co. (Arm & Hammer^®^, Ewing, NJ, USA), respectively. HPLC-grade acetic acid (99.5%), acetonitrile, and methanol as well as phosphate-buffered saline (PBS) tablets were obtained from Fischer Scientific (Pittsburgh, PA, USA). Water (HPLC-grade) was purchased from Macron Fine Chemicals (Center Valley, PA, USA). Commercial OchraTest WB immunoaffinity columns (IAC) were purchased from VICAM (Watertown, MA, USA) for sample cleanup and purification. OTA stock solution (100 mg/L) was prepared in methanol and further diluted with 50% methanol (methanol:water = 50:50; *v/v*) to prepare working standard solutions. All standard solutions were stored in amber vials at −20 °C.

### 5.2. Sample Preparation and Processing

Rice flour and oat flour were used to prepare porridges and their initial moisture contents were measured to be ca. 12.8% and 11.6% (wet weight basis, wb), respectively, by using Model HB43-S Halogen Moisture Analyzer (Mettler Toledo, Greifense, Switzerland). Fifty grams of rice or oat flour were spiked at levels of 20 μg/kg of flour by adding 1 mg/L OTA solution in methanol and then shaken intermittently for 2 h for even distribution. Each sample contaminated with OTA (50 g) was suspended in water (500 mL) to achieve 10% of solid basis. Then, sodium bicarbonate (0.5% and 1%), fructose (0.5%, 1%, 5%, and 10%), and a combination (sodium bicarbonate 0.5% + fructose 0.5%; *w/w*), were added to the flour and thoroughly mixed before the cooking process. A stainless steel pot filled with mineral oil was placed on a hot plate stirrer (Hei-Tec, Heidolph, Germany). Mineral oil was used as a heating medium and stirred with a magnetic bar to facilitate uniform distribution of the heat at 225 rpm. Then, another stainless steel pot for sample heating treatment was installed in the mineral oil chamber. Prepared rice- or oat-porridge samples were transferred into the pot and heated to 80–85 °C of center temperature then kept the temperature for 10 min for gelatinization. After the indirect steaming process, the samples were transferred onto stainless steel trays and then dried in an oven at 50 °C overnight. Dried samples were ground and then stored in plastic zipper bags at −20 °C until analyzed. OTA non-contaminated samples (or blanks) were used to compare with the normal operation of indirect steaming process in relation to the loss of OTA. We also confirmed that drying at 50 °C overnight did not change OTA levels in samples.

### 5.3. Analyses of Color and OTA

A spectrophotometer (Model CM-5, Konica Minolta Sensing Americas Inc., NJ, USA) was used to measure the color of ground samples according to the method used previously [24].

OTA concentrations in porridge samples were analyzed as described by Lee, Lee, and Ryu [24] with some modifications. In brief, a 5 g ground sample was extracted with 20 mL of 80% acetonitrile (acetonitrile:water = 80:20, *v/v*) for 30 min on a wrist action shaker (Burrell Scientific, Pittsburgh, PA, USA) followed by filtration (Whatman No. 1 filter paper). The filtrate was diluted five times with PBS and filtered again (Whatman No 1). For the sample cleanup, diluted extract (10 mL) was loaded and passed through an IAC column at a flow rate of about 2 mL/min, and the column was washed with PBS and water in sequence (10 mL each). OTA was then eluted with methanol (3 mL total at about 2 mL/min). The eluate collected in an amber vial was evaporated to dryness under a gentle stream of nitrogen at 50 °C using an aluminum heating block. The residue was reconstituted in 50% methanol (500 µL, methanol:water = 50:50; *v/v*). Finally, 10 µL of the purified extract was injected into HPLC consisted with a vacuum degasser, quaternary pump, autosampler, and fluorescence detector (Agilent 1260 Infinity system, Palo Alto, CA, USA). The chromatographic analysis was performed with a C18 column (Hypersil GOLD, 3 × 100 mm, particle size 1.9 µm, Thermo Scientific, Hudson, NH, USA) at room temperature under isocratic elution of a solvent mixture, i.e., 50% acetonitrile with 0.5% acetic acid, at a flow rate of 0.4 mL/min. OTA was detected at the wavelengths of 334 nm and 460 nm excitation and emission, respectively. For recovery, OTA was added to rice and oat flours at concentrations of 2, 10, and 20 μg/kg and then analyzed by following the same procedure described above.

### 5.4. Statistical Analysis

All experiments were replicated three times, and the Statistical Package for Social Sciences version 18.0 (SPSS Inc., Chicago, IL, USA) was used to analyze the data obtained from the experiments. The statistical differences among the treatments were determined by the one-way analysis of variance (ANOVA) with Tukey’s multiple range tests in addition to independent t-test (*p* < 0.05). All data are reported as means ± standard deviation (SD).

## Figures and Tables

**Table 1:** Effects of indirect steaming process and adding sodium bicarbonate, fructose, or combination of them on color parameters of retorted rice-based and oat-based porridge.

Sample	Additives		*L* ^1^	*A* ^2^	*B* ^3^	*ΔE* ^4^
	Type	Amount (%, *w*/*w*) ^5^				
Rice-based porridge	No additives		89.1 ± 1.4 ^a,6^	−0.7 ± 0.1 ^a^	8.6 ± 0.5 ^c^	1.9 ± 1.6 ^b^
	Sodium bicarbonate	0.5	85.9 ± 0.7 ^c^	−2.1 ± 0.4 ^c^	15.5 ± 1.0 ^a^	6.4 ± 1.7 ^a^
		1	86.7 ± 0.3 ^bc^	−3.1 ± 0.2 ^d^	17.2 ± 1.7 ^a^	8.0 ± 1.6 ^a^
	Fructose	0.5	89.2 ± 0.6 ^a^	−0.8 ± 0.1 ^a^	8.9 ± 0.6 ^c^	1.1 ± 0.5 ^b^
		1	87.8 ± 0.9 ^abc^	−0.8 ± 0.1 ^a^	9.7 ± 1.0 ^bc^	1.2 ± 1.3 ^b^
		5	87.1 ± 0.5 ^abc^	−0.9 ± 0.2 ^a^	10.8 ± 1.1 ^bc^	1.9 ± 1.3 ^b^
		10	88.7 ± 0.3 ^ab^	−0.6 ± 0.0 ^a^	10.6 ± 1.0 ^bc^	0.9 ± 0.5 ^b^
	Sodium bicarbonate + fructose	0.5 + 0.5	87.2 ± 0.6 ^abc^	−1.5 ± 0.1 ^b^	12.3 ± 1.0 ^b^	3.0 ± 0.5 ^b^
Oat-based porridge	No additives		72.3 ± 3.4 ^a^	3.1 ± 0.6 ^ab^	19.6 ± 2.3 ^NS^	3.1 ± 1.6 ^bc^
	Sodium bicarbonate	0.5	67.5 ± 2.3 ^ab^	2.7 ± 0.2 ^b^	18.5 ± 0.8	4.2 ± 2.3 ^bc^
		1	61.5 ± 1.3 ^c^	1.7 ± 0.2 ^c^	19.7 ± 0.8	10.2 ± 1.2 ^a^
	Fructose	0.5	72.9 ± 1.2 ^a^	2.8 ± 0.2 ^b^	18.4 ± 0.2	1.5 ± 1.2 ^c^
		1	70.6 ± 1.6 ^ab^	3.2 ± 0.2 ^ab^	18.4 ± 0.4	1.3 ± 0.9 ^c^
		5	67.6 ± 1.6 ^ab^	3.8 ± 0.2 ^a^	20.1 ± 0.5	4.2 ± 1.8 ^bc^
		10	71.2 ± 1.2 ^a^	3.2 ± 0.2 ^ab^	19.0 ± 0.2	1.0 ± 0.8 ^c^
	Combination of sodium bicarbonate and fructose	0.5 + 0.5	65.7 ± 1.7 ^bc^	2.8 ± 0.1 ^b^	18.1 ± 0.1	5.9 ± 1.4 ^b^

^1^ Degree of lightness (white + 100 → 0 black). ^2^ Degree of redness (red + 100 → −80 green). ^3^ Degree of yellowness (yellow + 70 → −80 blue). ^4^ Total color difference (*ΔE*) =$\sqrt{{\left(L-L_{0}\right)}^{2}+{\left(a-a_{0}\right)}^{2}+{\left(b-b_{0}\right)}^{2}}$, where, *L*, *a*, and *b* are values for the product, and *L*_0_, *a*_0_ and *b*_0_ are values for the raw composite grains. ^5^ Additive percent amount of rice/oat flour (*w*/*w*). ^6^ All values are mean ± SD and different superscript letters ^a,b,c,d^ indicate significant difference between groups within same commodity at *p* < 0.05 by Tukey’s multiple range tests. ^NS^ means not significant.

**Table 2:** Effects of added sodium bicarbonate and fructose on the reduction of ochratoxin A (OTA) in rice-based and oat-based porridges.

Additives		OTA Reduction (%) ^1^	
Type	Amount (%, *w*/*w*) ^2^	Rice-Based Porridge	Oat-Based Porridge
No additives		59.4 ± 1.9 ^c,3^	13.6 ± 1.2 ^e,^*
Sodium bicarbonate	0.5	78.1 ± 1.8 ^a^	57.7 ± 6.5 ^bc,^*
	1	68.7 ± 0.5 ^b^	72.6 ± 2.0 ^a^
Fructose	0.5	62.5 ± 1.2 ^bc^	47.3 ± 4.3 ^cd,^*
	1	80.7 ± 3.1 ^a^	69.3 ± 5.0 ^ab,^*
	5	66.1 ± 1.2 ^bc^	47.5 ± 7.8 ^cd,^*
	10	60.0 ± 5.3 ^c^	40.7 ± 1.2 ^d,^*
Combination of sodium bicarbonate and fructose	0.5 + 0.5	78.6 ± 1.0 ^a^	67.2 ± 5.9 ^ab^

^1^ OTA reduction (%) = (1 − OTA concentration in retorted sample/OTA concentration in unsteamed sample) × 100; moisture adjusted. ^2^ Additive percent amount of rice/oat flour (*w*/*w*). ^3^ All values are mean ± SD and different superscript letters ^a,b,c,d,e^ indicate significant difference between groups within same commodity at *p* < 0.05 by Tukey’s multiple range tests and star symbols * indicate significant difference between same treatment groups at *p* < 0.05 by independent samples *t*-test.

## Data Availability

The data presented in this study are available in article here.
